# A Realist Review Protocol on the Contribution of  Mental Health First Aider Programmes to Staff Wellbeing within Hospital and associated healthcare settings

**DOI:** 10.12688/hrbopenres.14318.2

**Published:** 2026-04-03

**Authors:** Dolores Donegan, Sean Paul Teeling, Martin McNamara

**Affiliations:** 1 UCD School of Nursing and Midwifery and Health Systems, University College Dublin, Ireland, Dublin, Ireland; 2 Centre for Person-Centered Practice Research, Queen Margaret University School of Health Sciences, Musselburgh, Scotland, EH21 6UU, UK; 3 UCD Centre for Interdisciplinary Research, Education and Innovation in Health Systems, School of Nursing, Midwifery & Health Systems, University College Dublin., Dublin, D04 V1W8, Ireland

**Keywords:** Realist Review Protocol, Hospital Staff, Mental Health First Aid, Staff Wellbeing, Healthcare Workers.

## Abstract

**Background:**

Staff wellbeing is a growing concerns in healthcare settings, where increased workload, emotional demand, and organisational pressures contribute to stress, burnout, and reduced psychological safety. Mental Health First Aid (MHFA) training is an evidence-based programme designed to support early recognition of mental distress, provide initial reassurance, and facilitate signposting to appropriate help. Its specific contribution to staff wellbeing within hospital and associated healthcare settings, however, remains underexplored. This paper presents a protocol for a realist review that will examine how, why, and in what contexts an MHFA programme may support staff wellbeing within hospital and associated healthcare settings.

**Methods:**

This protocol for a realist review will follow RAMESES standards to examine the contribution of MHFA training to staff health and wellbeing. The review will proceed through five stages: (1) clarifying the scope of the review; (2) developing initial programme theories; (3) searching for evidence; (4) extracting and synthesising data using realist logic; and (5) refining the programme theories. An expert panel will be convened to support the development and refinement of the programme theories as the review progresses. Conclusions This realist review protocol will guide the development of context–mechanism–outcome (CMO) configurations to explain how MHFA training may operate within different hospital contexts. The resulting programme theories may contribute to organisational strategy, leadership practice, and policy decisions aimed at improving staff wellbeing in acute hospital settings.

**Table 1.** PICO (Population, Intervention, Context, Outcomes) framework.

PICO element	Key concept	Inclusion/Exclusion criteria
Population	Healthcare Staff in hospitals and related healthcare settings	Inclusion: Healthcare Staff in hospitals and related healthcare settings who have participated in, delivered, or been exposed to MHFA programmes Inclusion: Studies from adjacent healthcare contexts (e.g., community or specialist settings) will be considered where they provide transferable explanatory insight relevant to hospital staff wellbeing Exclusion: Healthcare Staff in hospitals and related healthcare settings who have not participated in, delivered, or been exposed to MHFA programmes
Intervention	Mental Health First aid Training and associated Implementation approaches	Included: Mental Health First aid Training and associated Implementation approaches
Context	Time	Inclusion: Publications 2010–2026. Limiting the search will support access to more recent date research articles
	Language	Inclusion: Publications in English
	Hospitals and related healthcare organisational settings	Inclusion: Hospitals and related healthcare organisational settings, including leadership support, team culture, psychological safety, stigma, and implementation conditions Inclusion: Studies from adjacent healthcare contexts will be considered where they provide transferable explanatory insights
Outcome	Proximal	Confidence to provide peer support
		Reduce mental health stigma
		Improved recognition of mental distress
	Intermediate	Help seeking behaviours
		Psychological safety
		Perceived Organisational Support
	Distal	Patterns of absenteeism or retention
	Use of Staff Supports	Broader Impacts on staff wellbeing

## Introduction

Mental health is described by the World Health Organisation ( WHO, 2019) as a state of mental well-being that enables people to cope with the stresses of life, realise their abilities, learn well and work well, and contribute to their community. It has intrinsic and instrumental value and is integral to our well-being. Increasing demands on hospitals have led to concerns around the working conditions and wellbeing of staff ( Kinman & Teoh, 2018). Healthcare staff typically access mental health support through an Employee Assistance Programme (EAP), a work-based service that helps staff and organizations with personal, work-related, and psychosocial issues. Workplace stress “particularly among frontline staff” is the number one issue raised by healthcare staff within the EAP ( Lynch, 2023).

In this review, staff wellbeing will be conceptualised as the primary outcome and will refer to psychological wellbeing and related workplace experiences, including mental distress, burnout-related strain, psychological safety, and perceived organisational support. Workplace culture will be considered a contextual and relational influence that may enable or constrain the mechanisms through which MHFA affects staff wellbeing, rather than a standalone outcome.

Mental ill health imposes significant economic burdens, manifesting in various ways such as elevated expenses due to absenteeism and staff turnover, as well as heightened pressure on healthcare service ( Cook *et al.*, 2024). Friends and family may notice early signs of mental illness but often don’t know how to help, resulting in delayed access to treatment. Programmes such as Mental Health First Aid (MHFA) offer evidence-based knowledge and skills to enhance engagement and prompt support. Insufficient focus on preventative initiatives has been posited as an explanation for sustained mental ill health prevalence rates in several countries, despite increased mental health expenditure and service provision ( Jorm *et al.*, 2017; Meadows *et al.*, 2019). Delays in seeking care can exacerbate mental ill health conditions leading to worse outcomes and reduced quality of life ( Ahad *et al.*, 2023). Through MHFA training the whole hospital community can assist formal mental health services in early intervention for mental health disorders ( Kitchener & Jorm, 2008 p. 59).

MHFA programmes are internationally recognised, evidence-based, accredited training courses designed to inform individuals about how to support someone experiencing mental ill health. This study aims to identify which aspects of the MHFA programme benefit whom, under what conditions, and how best to enhance hospital staff wellbeing.

## Use of realist inquiry

Realist inquiry aims to understand “what works, for whom, under what circumstances, and why ( Pawson *et al.*, 2005; Pawson, 2006; Teeling *et al.*, 2021a; Teeling *et al.*, 2021b). Realist inquiry goes beyond exploring surface-level inputs and outputs by discerning how the resources of a specific intervention evoke and interact with stakeholders’ psychosocial responses and trigger mechanisms (M) that generate intervention outcomes (O) in specific contexts (C) ( de Brun *et al.*, 2020). Researchers develop a Context-Mechanism-Outcome (CMO) hypothesis to predict which mechanisms operate in different contexts and the resulting outcomes ( Dalkin *et al.*, 2015). This configuration is ideal for exploring the context-sensitivity of the impact of MHFA programmes on staff wellbeing in hospital and associated healthcare networks. For example, a potential early CMO Hypothesis could be, *in contexts where senior leadership visibly supports MHFA participation and staff have protected time to attend training (C), the programme may trigger increased confidence and perceived organisational support among staff (M), leading to earlier peer signposting and help-seeking behaviours (O).* Such provisional hypotheses will be tested and refined during the review.

This realist review protocol seeks to understand how, why, for whom, and under what circumstances MHFA programmes can influence staff wellbeing in hospital and associated healthcare settings. While existing evidence highlights the potential benefits of MHFA programme, its effectiveness is likely shaped by contextual factors, such as organisational culture, leadership support, and workforce dynamics. ( Kitchener & Jorm, 2008). By exploring the mechanisms through which MHFA operates and the conditions that enable or hinder its success, this research seeks to provide a nuanced understanding of its impact.

The research question guiding the realist review described in this protocol is.

How, why, for whom, and under what circumstances do MHFA programmes influence staff wellbeing within hospital and associated healthcare settings?

The RAMESES guidelines provide standards to improve the rigour and comparability of realist and meta-narrative reviews ( Greenhalgh *et al.*, 2015). A five-stage structured methodology consistent with the RAMESES guidelines has been developed ( Wong *et al.*, 2017) which has been utilised to inform this review:

1. Define the scope of the review and develop initial theories.
2. Develop the search strategy.
3. Review primary studies and extract data.
4. Synthesise evidence and develop conclusions.
5. Refine theory iteratively and disseminate findings.

### Define the scope of the review and develop initial theories

The development of Candidate Programme Theories (CPTs) in a realist review provides a framework for analysing the causal mechanisms that may generate outcomes within specific contexts ( Teeling *et al.*, 2021b). Developing CPTs will involve identifying possible explanations for how the MHFA programme may work, for whom, and under what circumstances. This theoretical mapping will draw on a scoping review of existing literature, expert insights, and early conceptual thinking to form initial hypotheses about how the intervention may operate ( Keown *et al.*, 2024).

Realist “hunches” are initial, theory-informed assumptions about how an intervention might work, for whom, and in what contexts ( Pawson, 2013). These early ideas will guide the identification of potential mechanisms and relevant contextual conditions. Academic supervision will support the assessment of these developing ideas, ensuring their plausibility and relevance to the MHFA programme ( Wong *et al.*, 2013).

To ensure practical and contextual relevance, a Local Reference Group (LRG) will be convened. The LRG will comprise MHFA programme participants, accredited MHFA instructors involved in delivery, and staff with responsibility for organisational wellbeing initiatives within hospital and associated healthcare settings. Membership will be purposively selected to ensure representation of delivery, participation, and implementation perspectives ( Wong *et al.*, 2016). Their insights will help refine developing CPTs so that emerging theories remain grounded in real-world experience and practice.

A preliminary scoping of the literature will further support the development of CPTs. Searches will include electronic databases (PubMed, Web of Science, PsycINFO, CINAHL, EMBASE, Cochrane Library), grey literature, and organisational sources such as the MHFA International website, using keywords related to realist review, healthcare staff, staff wellbeing, mental health, and MHFA ( Booth, 2016).

Once CPTs have been developed in conjunction with the LRG and the scoping literature, they will be presented to an expert review panel for adjudication. The expert panel will comprise academic researchers with recognised expertise in realist review and organisational wellbeing research, alongside accredited MHFA instructors with experience of programme delivery and implementation in healthcare. Panel members will be selected based on demonstrated methodological and subject expertise to support credible adjudication of developing programme theories ( Wong *et al.*, 2016). The panel will confirm, refute, or refine the developing CPTs to strengthen their methodological rigour and practical relevance.

Following expert adjudication, the refined CPTs will become the Initial Programme Theories (IPTs). These IPTs will inform the next stages of the review, including refinement of the search strategy, development of inclusion and exclusion criteria, data extraction, and synthesis. As evidence is gathered, the IPTs will be iteratively tested and refined in line with realist principles ( Dalkin *et al.*, 2015; Wong *et al.*, 2013).

This process will ensure the development of a coherent set of programme theories that will guide the remainder of the realist review.

### Develop the search strategy

A comprehensive and theory-informed search strategy will be essential to support the development and refinement of programme theories for this realist review. The search strategy will follow RAMESES guidance, which emphasises iterative, purposive searching aligned with the evolving focus of the review ( Wong *et al.*, 2013; Wong *et al.*, 2016).

Searches will be conducted across multiple electronic databases, including PubMed, Web of Science, PsycINFO, CINAHL, EMBASE, and the Cochrane Library. Additional sources will include grey literature, organisational websites such as MHFA International, citation searching, and the reference lists of included studies ( Booth, 2016). The support of a university librarian will ensure that search processes are systematic, comprehensive, and appropriately targeted.

A PICO (Population, Intervention, Context, Outcomes) framework will be used to structure the development of search terms and guide inclusion and exclusion criteria ( Methley *et al.*, 2014; Schardt *et al.*, 2007). The PICO criteria have been developed based on the research question, preliminary scoping of the literature, and anticipated areas of theoretical interest ( Booth, 2016; Pawson, 2013). This approach allows the PICO inclusion and exclusion criteria to be refined as the Candidate Programme Theories (CPTs) are developed and formalised, prior to their adjudication by the Expert Panel and progression to Initial Programme Theories (IPTs). These IPTs will then be tested through the realist review to develop Programme Theories (PTs). The PICO framework ( Table 1) will therefore serve as a guide for identifying relevant evidence. It will help ensure that search terms capture literature related to MHFA training, staff wellbeing, contextual influences on psychological support interventions, and implementation processes within acute hospital settings.

### Eligibility approach

Studies will be included based on their contribution to programme theory development (contexts, mechanisms, outcomes), rather than requiring predefined quantitative indicators.

In line with realist methodology, outcomes will be conceptualised at three levels ( Pawson & Tilley, 1997; Wong et al., 2013). Proximal outcomes will refer to immediate changes such as increased confidence or reduced stigma. Intermediate outcomes will include help-seeking behaviour and psychological safety. Distal outcomes will refer to broader organisational indicators such as absenteeism patterns or retention. This layered structure will guide programme theory development.

Consistent with realist methodology, searching will be iterative and theory-led rather than linear ( Pawson, 2013; Wong et al., 2013). The PICO framework will be used to structure the initial search strategy and enhance transparency, but it will not function as a rigid eligibility filter. As programme theories evolve, further purposive searches may be undertaken to explore emerging contextual or mechanistic explanations. Study inclusion will therefore be guided by relevance, rigour, and richness of explanatory data ( Rycroft-Malone et al., 2016).

These PICO criteria will provide the foundation for identifying potentially relevant evidence. As the Candidate Programme Theories (CPTs) are formalised and progress to Initial Programme Theories (IPTs), the selection of studies and the approach to data extraction will evolve to ensure alignment with the developing programme theories. This iterative refinement of searching and evidence selection is consistent with realist methodology, which emphasises the need for theory-driven and adaptive approaches to evidence gathering ( Wong *et al.*, 2013). The next section outlines how studies will be screened and how data will be extracted to support this realist review.

### Review primary studies and extract data

Documents retrieved will be chosen based on their relevance to the research question and their potential to support the development of IPTs. These documents will form a robust collection of papers, essential for a theory-driven realist review to further refine the IPTs ( Greenhalgh *et al.*, 2011). The selection of studies will be guided by the realist criteria of relevance, richness, and rigour ( Booth *et al.*, 2013). Relevance is assessed by whether a study can contribute to building or testing a programme theory; richness refers to depth, detail, and complexity of data, which includes deep descriptions, contextual understanding, and multifaceted insights into human experiences rather than surface-level information; and rigour refers to the credibility of the methods used to collect the data ( Rycroft-Malone *et al.*, 2016; Wong *et al.*, 2013). These criteria will collectively determine which literature is included in the review.

Data extraction will involve three steps: initial screening by title and abstract, full-text retrieval, and quality appraisal. Titles and abstracts will be reviewed in duplicate by Authors 1 and 2 using predefined criteria. Documents passing this stage will then be adjudicated for richness and rigour by Author 3. Any discrepancies will be resolved through discussion among all three authors, in line with RAMESES guidelines ( Wong *et al.*, 2013).

### Synthesize evidence and develop conclusions

Search results will be imported into Covidence, an online software for managing and screening systematic search results, which is freely available to staff and students of University College Dublin. Rayyan is an alternative that is freely available. The analysis and synthesis process will follow the stages outlined by Rycroft-Malone *et al.* (2012), using an iterative, theory-driven approach appropriate for realist methodology. These stages will include:

• Organising extracted data into evidence tables;
• Theming by individual reviewers;
• Comparing reviewers’ themes for each article and developing chains of inference from the identified themes;
• Linking chains of inference across articles and tracking how evidence contributes to emerging theory;
• Formulating hypotheses represented as linked context–mechanism–outcome (CMO) chains.

Retroductive theorising will be used to synthesise data ( Mukumbang *et al.*, 2021). Retroduction involves an iterative cycle of inductive reasoning, deductive reasoning, and abduction to identify the underlying causal forces that may explain how the MHFA programme works ( Gilmore *et al.*, 2019). Endnote software will be used to record the references from search results and any additional references included during the review, and the review stages will be revisited as necessary to ensure comprehensive evidence gathering and to support theory saturation. EndNote is freely available to staff and students of University College Dublin, Zotero is an alternative which is freely available.

### Refine theory iteratively and disseminate findings

The refined Initial Programme Theories (IPTs) derived from the realist review will undergo iterative evaluation and feedback from the expert panel to ensure accurate interpretation and explanation of the findings ( Pawson *et al.*, 2005). This engagement will enhance the robustness, contextual sensitivity, and real-world relevance of the resulting programme theories, reflecting the complexity of MHFA implementation in acute hospital settings ( Wong *et al.*, 2016).

The outcomes of the realist review will be reported in accordance with the RAMESES II reporting guidelines ( Wong, 2018). Dissemination will include publication in peer-reviewed journals, presentations at healthcare and academic conferences, and engagement with key organisational stakeholders.

This realist review protocol is currently under consideration by the PROSPERO Editorial Committee, and a registration number will be added once approval is confirmed. Ethical approval is not required for this review because it synthesises published literature and involves no primary data collection. Ethical approval will be sought for any subsequent realist evaluation involving new data collection.

The research team includes members with experience in healthcare improvement and organisational wellbeing research, as well as familiarity with hospital-based wellbeing initiatives. This experience provides contextual insight into the organisational environments in which such initiatives are implemented, but it may also shape how evidence is interpreted. In keeping with realist methodology, the research team will engage critically with their assumptions throughout the research process, recognizing that programme theory development involves interpretive judgement ( Pawson, 2013). Reflexive discussion within the research team, alongside expert panel adjudication and stakeholder engagement during programme theory refinement, will support methodological rigour and help minimise the risk of interpretive bias ( Teeling *et al.*, 2021b). These processes ensure that emerging interpretations are challenged, debated and tested against multiple perspectives during theory development.

## Conclusion

The realist review outlined in this protocol aims to explore how, why, for whom, and under what circumstances the MHFA programme may promote staff wellbeing in hospital and associated healthcare settings. By adopting a critical realist approach and utilising realist inquiry, this review will seek to:

• Determine for whom the MHFA programme works or does not work, and why;
• Identify the contexts in which the MHFA programme may be effective or ineffective, and the reasons for these variations;
• Uncover the mechanisms through which the MHFA programme may achieve its intended outcomes in specific contexts;
• Evaluate the anticipated and unanticipated outcomes of the MHFA programme for staff wellbeing.

This approach will support the identification not only of potentially effective strategies, but also of the underlying causal processes and contextual conditions that may shape their success, providing a robust framework for understanding mechanisms in complex organisational environments ( Pawson & Tilley, 1997). It is anticipated that the findings from the realist review described in this protocol will contribute to understanding how MHFA training may support staff wellbeing in hospital and associated healthcare settings.

### Systematic review registration

This protocol is currently undergoing review by the PROSPERO Editorial committee, and registration number will be added as soon as available.

## Data Availability

No data are associated with this article. Donegan *et al.* (2025). Prisma-P 2015 Checklist for realist review protocol: A Realist Review Protocol on the Contribution of Mental Health First Aider Programmes to Staff Wellbeing within Hospital and associated healthcare settings. https://doi.org/10.6084/m9.figshare.30815501 Data are available under the terms of the Creative Commons Attribution 4.0 International license (CC-BY 4.0). The Realist Review will be informed by the RAMESES (Realist and Meta-narrative Evidence Synthesis: Evolving Standards) project.

## References

[ref1] Ahad AA Sanchez-Gonzalez M Junquera P : Understanding and addressing mental health stigma across cultures for improving psychiatric care: a narrative review. *Cureus.* 2023;15(5):e39549. , 10.7759/cureus.39549 37250612 PMC10220277

[ref2] Booth A : Searching for qualitative research for inclusion in systematic reviews: a structured methodological review. *Syst Rev.* 2016;5:74. 10.1186/s13643-016-0249-x 27145932 PMC4855695

[ref3] Booth A Harris J Croot E : Towards a methodology for cluster searching to provide conceptual and contextual "richness" for systematic reviews of complex interventions: case study (CLUSTER). *BMC Med Res Methodol.* 2013;13:118. 10.1186/1471-2288-13-118 24073615 PMC3819734

[ref4] Cook A Keyte R Sprawson I : Mental Health First Aid experiences: a qualitive investigation into the emotional impact of Mental Health First Aid responsibilities and the significance of self-compassion. *SN Social Sciences.* 2024;4:171. 10.1007/s43545-024-00962-1

[ref5] Dalkin SM Greenhalgh J Jones D : What's in a mechanism? Development of a key concept in realist evaluation. *Implement Sci.* 2015;10(1):49. 10.1186/s13012-015-0237-x 25885787 PMC4408605

[ref6] De Brun A Rogers L O’Shea M : Understanding the impact of a collective leadership intervention on team working and safety culture in healthcare teams: a realist evaluation protocol [version 2; peer review: 2 approved]. *HRB Open Res.* 2020;2:5. 10.12688/hrbopenres.12860.2 32296745 PMC7140778

[ref7] Donegan D Teeling SP McNamara M : PRISMA-P 2015 checklist for realist review protocol: a realist review protocol on the contribution of a Mental Health First Aider programme to staff health and wellbeing within an Irish hospital network. *figshare.* 2025. 10.6084/m9.figshare.30815501.v1 PMC13100600 42027650

[ref8] Gilmore B McAuliffe E Power J : Data analysis and synthesis within a realist evaluation: toward more transparent methodological approaches. *Int J Qual. Methods.* 2019;18:18. 10.1177/1609406919859754

[ref9] Greenhalgh T Wong G Jagosh J : Protocol—the RAMESES II study: developing guidance and reporting standards for realist evaluation. *BMJ Open.* 2015;5(8):e008567. 10.1136/bmjopen-2015-008567 PMC4538260 26238395

[ref10] Greenhalgh T Wong G Westhorp G : Protocol-Realist and Metanarrative Evidence Synthesis: Evolving Standards (RAMESES). *BMC Med Res Methodol.* 2011;11:115. 10.1186/1471-2288-11-115 21843376 PMC3173389

[ref11] Jorm AF Patten SB Brugha TS : Has increased provision of treatment reduced the prevalence of common mental disorders? Review of the evidence from four countries. *World Psychiatry.* 2017;16(1):90–99. 10.1002/wps.20388 28127925 PMC5269479

[ref12] Keown AM Teeling SP Mcnamara M : The contribution of leaders' and managers' attributes, values, principles, and behaviours to the sustainable implementation of lean in healthcare: a realist review protocol [version 1; peer review: 2 approved]. *HRB Open Res.* 2024;7:54. 10.12688/hrbopenres.13933.1 39897597 PMC11786105

[ref13] Kinman A Teoh KRH : What could make a difference to mental health of UK doctors? A review of the research evidence. 2018. Reference Source

[ref14] Kitchener BA Jorm AF : Mental Health First Aid: an international programme for early intervention. *Early Interv Psychiatry.* 2008;2(1):55–61. 10.1111/j.1751-7893.2007.00056.x 21352133

[ref15] Lynch D : Workplace stress is ‘number one issue’ raised with HSE Employee Assistance Programme. The medical independent, {sourced 30th October 2025},2023. Reference Source

[ref16] Meadows GN Prodan A Patten S : Resolving the paradox of increased mental health expenditure and stable prevalence. *Aust N Z J Psychiatry.* 2019;53(9):844–850. 10.1177/0004867419857821 31238699 PMC6724452

[ref17] Methley AM Campbell S Chew-Graham C PICO : PICOS and SPIDER: a comparison study of specificity and sensitivity in three search tools for qualitative systematic reviews. *BMC Health Serv Res.* 2014;14:579. 10.1186/s12913-014-0579-0 25413154 PMC4310146

[ref18] Mukumbang FC Kabongo EM Eastwood JG : Examining the application of retroductive theorizing in realist-informed studies. *Int J Qual Methods.* 2021;20(1):1–14. 10.1177/16094069211053516

[ref19] Pawson R : Digging for nuggets: how ‘Bad’ research can yield ‘Good’ evidence. *Int J Soc Res Methodol.* 2006;9(2):127–142. 10.1080/13645570600595314

[ref20] Pawson R : *The science of evaluation: a realist manifesto.* London: Sage;2013. 10.4135/9781473913820

[ref21] Pawson R Greenhalgh T Harvey G : Realist review -- a new method of systematic review designed for complex policy interventions. *J Health Serv Res Policy.* 2005;10(Suppl 1):21–34. 10.1258/1355819054308530 16053581

[ref22] Pawson R Tilley N : An introduction to scientific realist evaluation. *Sage, London*; 1997. 10.4135/9781483348896.n29

[ref23] Rycroft-Malone J Burton CR Bucknall T Graham ID Hutchinson AM Stacey D : Collaboration and co-production of knowledge in healthcare: opportunities and challenges. *Int J Health Policy Manag.* 2016;5(4):221–223. 27239867 10.15171/ijhpm.2016.08 PMC4818986

[ref24] Rycroft-Malone J McCormack B Hutchinson AM : Realist synthesis: illustrating the method for implementation research. *Implement Sci.* 2012;7:33. 10.1186/1748-5908-7-33 22515663 PMC3514310

[ref25] Schardt C Adams MB Owens T : Utilization of the PICO framework to improve searching PubMed for clinical questions. *BMC Med Inform Decis Mak.* 2007;7(1):16. 10.1186/1472-6947-7-16 17573961 PMC1904193

[ref26] Teeling SP Davies C Barnard M : A rapid realist review of quality care process metrics implementation in nursing and midwifery practice. *Int J Environ Res Public Health.* 2021a;18(22):11932. 10.3390/ijerph182211932 34831694 PMC8621300

[ref27] Teeling SP Dewing J : Baldie D: A realist inquiry to identify the contribution of Lean Six Sigma to person-centred care and cultures. *Int J Environ Res Public Health.* 2021b;18(19):10427. 10.3390/ijerph181910427 34639727 PMC8507723

[ref28] Wong G : Realist reviews in health policy and systems research. Evidence synthesis for health policy and systems: a methods guide. *World Health Organization*; 2018. 33877749

[ref29] Wong G Greenhalgh T Westhorp G : RAMESES publication standards: meta-narrative reviews. *BMC Med.* 2013;11:20. 10.1186/1741-7015-11-20 23360661 PMC3558334

[ref30] Wong G Westhorp G Greenhalgh J : Quality and reporting standards, resources, training materials and information for realist evaluation: the RAMESES II project. *Health Serv Deliv Res.* 2017;5(28):1–108. 10.3310/hsdr05280 29072890

[ref31] Wong G Westhorp G Manzano A : RAMESES II reporting standards for realist evaluations. *BMC Med.* 2016;14(1):96. 10.1186/s12916-016-0643-1 27342217 PMC4920991

[ref32] World Health Organisation : mhGAP community toolkit: field test version. Geneva: World Health Organization.2019; [accessed 14 Dec 2024]. Reference Source

