# Supplementary material for: A Realist Review Protocol on the Contribution of  Mental Health First Aider Programmes to Staff Wellbeing within Hospital and associated healthcare settings
Source: HRB Open Res. 2026 Apr 3;9:7. Originally published 2026 Jan 9. [Version 2] doi: 10.12688/hrbopenres.14318.2 (PMC13100600; doi:10.12688/hrbopenres.14318.2)
Supplement: Peer review history

# Reviewer report: Reviewer response for version 2

**DOI:** 10.21956/hrbopenres.15879.r54456

**Published:** 2026-04-21

**Authors:** Mary Lavelle

**Affiliations:** 1 Queen's University Belfast, Belfast, Northern Ireland, UK

The authors have clearly considered the feedback provided and have improved the clarity of the manuscript. I am happy to approve this manuscript at this stage.

Is the study design appropriate for the research question?

Partly

Is the rationale for, and objectives of, the study clearly described?

Yes

Are sufficient details of the methods provided to allow replication by others?

Partly

Are the datasets clearly presented in a useable and accessible format?

Not applicable

Reviewer Expertise:

Mental Health research, both qualitative and quantitative methods. I have not conducted a realist review.

I confirm that I have read this submission and believe that I have an appropriate level of expertise to confirm that it is of an acceptable scientific standard.

# Reviewer report: Reviewer response for version 2

**DOI:** 10.21956/hrbopenres.15879.r54455

**Published:** 2026-04-07

**Authors:** Karel Desmet

**Affiliations:** 1 General Hospital Ostend, Ostend, Belgium; 2 Ghent University, Ghent, Flanders, Belgium

The authors are to be commended for the rigor and diligence demonstrated throughout the revision process. Their responses are consistently comprehensive and well-articulated, reflecting a thorough engagement with the comments. Moreover, the revisions made to the manuscript are both appropriate and substantively improve its clarity and scholarly contribution. The authors also demonstrate a commendable level of critical reflection, carefully considering the implications of the feedback and transparently justifying their choices. Overall, this iterative process has clearly strengthened the manuscript.

Is the study design appropriate for the research question?

Yes

Is the rationale for, and objectives of, the study clearly described?

Yes

Are sufficient details of the methods provided to allow replication by others?

Partly

Are the datasets clearly presented in a useable and accessible format?

Yes

Reviewer Expertise:

mental health research

I confirm that I have read this submission and believe that I have an appropriate level of expertise to confirm that it is of an acceptable scientific standard.

# Reviewer report: Reviewer response for version 1

**DOI:** 10.21956/hrbopenres.15761.r53376

**Published:** 2026-02-17

**Authors:** Mary Lavelle

**Affiliations:** 1 Queen's University Belfast, Belfast, Northern Ireland, UK

This manuscript presents a protocol for a realist review examining how, why, and in what contexts Mental Health First Aid (MHFA) programmes may support staff health and wellbeing within a hospital network. The rationale for undertaking a realist approach is clearly articulated and well justified. There are some points that require further clarification, which I have summarised below. 

 The title refers specifically to the contribution of the programme within an Irish Hospital Network, yet the proposed review does not appear to be limited to Irish settings. This may create some ambiguity about the scope of the review.

 Different terms are used throughout the manuscript when referring to the primary outcome, including “staff health and wellbeing,” “staff wellbeing,” and, in the conclusion, “workplace culture.” It would strengthen the protocol to define more precisely the core outcome(s) of interest and to use consistent terminology throughout. If workplace culture is intended as a key outcome, this should be made explicit earlier in the manuscript. Alternatively, if workplace culture is being conceptualised as a contextual factor or mechanism within the realist framework, this should be clearly articulated.

 Relatedly, in Table 1 (PICO), the outcomes section appears relatively narrow and does not include reference to workplace culture. Could the PICO be too rigid for this type of review? If workplace culture is a central concept within the review, consideration should be given to whether it should be incorporated into the eligibility criteria and reflected explicitly within the outcomes (or context/mechanism) component of the framework.

 Overall, greater conceptual coherence between the title, stated aims, search strategy, and conclusion would strengthen the clarity and rigour of the protocol.

Is the study design appropriate for the research question?

Partly

Is the rationale for, and objectives of, the study clearly described?

Yes

Are sufficient details of the methods provided to allow replication by others?

Partly

Are the datasets clearly presented in a useable and accessible format?

Not applicable

Reviewer Expertise:

Mental Health research, both qualitative and quantitative methods. I have not conducted a realist review.

I confirm that I have read this submission and believe that I have an appropriate level of expertise to confirm that it is of an acceptable scientific standard, however I have significant reservations, as outlined above.

# Response

**Published:** 2026-03-16

**Authors:** Sean Paul Teeling

Dear Dr Lavelle,

 Thank you for taking the time to review the protocol and for the thoughtful and constructive comments provided. Your observations were particularly helpful in strengthening the clarity of the title, improving conceptual consistency across the manuscript, and refining the framing of the PICO to better reflect the explanatory focus of the review.

 In response to your observation that the title referred to an Irish Hospital Network while the review itself was not restricted to Ireland, the title and related sections of the manuscript have been revised to remove this ambiguity. The title now focuses on staff wellbeing within a hospital network rather than specifying a national context. This better reflects the scope of the realist review, which draws on international evidence to develop programme theories. The subsequent realist evaluation phase of the wider study will be conducted within Irish hospital sites; however, the review itself is not geographically restricted. Related terminology within the abstract has also been revised to ensure consistency, including replacing “Staff Health and Wellbeing” with “Staff Wellbeing” and removing the reference to an “Irish Hospital Network” (Title section, Page 1; Abstract).

 Your comment regarding inconsistent terminology across the manuscript was also extremely helpful. The protocol has been revised to ensure consistent use of the term “staff wellbeing” throughout the title, aims, search strategy and conclusion. In addition, “workplace culture” is now positioned more clearly as a contextual influence that may shape how programme mechanisms operate within organisational settings rather than being presented as a standalone outcome. These revisions strengthen conceptual alignment across the protocol and improve clarity regarding the key concepts underpinning the review.

 Finally, we appreciate your observation that the original PICO appeared overly rigid and did not sufficiently capture the role of workplace culture. In response, the PICO has been revised to better reflect the explanatory orientation of realist methodology. Culture is now positioned within the context component of the framework, recognising its influence on how programme mechanisms may operate in organisational environments. Outcomes are also conceptualised in a layered manner rather than through predefined metric thresholds, allowing for consideration of proximal, intermediate and distal outcomes. The text has also been revised to clarify that study inclusion will be guided by theory development and explanatory relevance rather than by predefined performance indicators (see Line 125).

 All revisions have been highlighted in yellow in the revised manuscript, and the relevant line numbers have been provided above to assist in locating the changes. Your comments have been extremely helpful in strengthening the conceptual clarity and methodological coherence of the protocol.

# Reviewer report: Reviewer response for version 1

**DOI:** 10.21956/hrbopenres.15761.r53127

**Published:** 2026-02-09

**Authors:** Karel Desmet

**Affiliations:** 1 General Hospital Ostend, Ostend, Belgium; 2 Ghent University, Ghent, Flanders, Belgium

Thank you for submitting this manuscript, which addresses a highly relevant topic in a rapidly evolving healthcare landscape. The protocol paper presents a valuable contribution to the field and is well-structured. The focus on mechanisms, contextual contingencies, and implementation processes is well aligned with the complexity of workplace mental health interventions. However, several clarifications and expansions could be strengthened to enhance its precision, clarity, methodological coherence and readability.

 The authors convincingly justify the use of realist inquiry. The emphasis on context–mechanism–outcome (CMO) configurations and retroductive theorising appropriately reflects the complexity of MHFA as a social and organisational intervention rather than a simple training “dose–response” model. The intention to move beyond effectiveness questions toward explanatory theory building is a clear strength. The integration of local stakeholders (Local Reference Group and expert panel) is particularly valuable and consistent with realist principles of theory refinement through practitioner knowledge. Is it possible to add more information about the MFA trainers, participants and expert trainers. For a non-UK reader this is not clear. Please add some more information about the experts to enhance validity and relevance.

 Some terms could be defined more precisely. For instance, ‘staff wellbeing’ is used broadly. Clarifying whether this refers to psychological wellbeing, burnout, mental distress, organizational outcomes, or a combination would sharpen the analytical focus.

 Although realist reviews are not outcome-centric in the traditional sense, the protocol lists several operational indicators (e.g., absenteeism rates, health checks, percentage trained). These metrics risk framing MHFA in primarily instrumental or managerial terms. It may be helpful to: distinguish between proximal outcomes (e.g., confidence to provide peer support, stigma reduction), intermediate outcomes (e.g., help-seeking behaviour, psychological safety), distal organisational outcomes (e.g., absenteeism, retention). This layered outcome logic could strengthen the program theory and prevent conflating mechanisms with endpoints.

 The use of a PICO structure to guide searching is understandable for transparency, but it sits somewhat uneasily with realist logic. PICO tends to imply intervention–outcome testing (and pragmatically used for systematic reviews), whereas realist synthesis is theory-led and mechanism-focused. Consider clarifying inclusion decisions more theory-driven. Without this clarification, the criteria (e.g., limiting to staff who participated MHFA, or requiring certain outcomes) may inadvertently exclude conceptually rich studies that inform mechanisms but do not report predefined metrics.

 Restricting settings to ‘acute hospitals participating in MHFA training for two years or more’ may unintentionally narrow the explanatory potential of the review. Realist reviews often benefit from conceptual heterogeneity, including studies from adjacent contexts that illuminate mechanisms. The authors might reflect on whether: shorter implementations, other healthcare or organisational settings, or broader workplace MHFA applications could provide transferable insights into mechanisms. A slightly more permissive approach could enrich theory development.

 The description of synthesis is appropriate but could be made more explicit. For example: How will CMO configurations be documented (software, templates, coding framework)? Will coding be double-checked or discussed collaboratively? How will disagreements about inferred mechanisms be resolved? Providing these operational details would enhance methodological transparency.

 The protocol mentions “realist hunches,” CPTs, and IPTs, which is consistent with realist language. However, the process could be described more concretely. A brief illustrative example of a potential CMO hypothesis (even hypothetical) would help readers unfamiliar with realist work to understand how theories will be articulated and refined.

 Minor suggestions: Ensure consistent terminology (MHFA programme vs training vs intervention). Check citation formatting and typographical accuracy. Avoid over-assertive claims about “evidence-based” status unless clearly specified. Consider a short reflexivity statement describing the team’s positioning, particularly given local implementation links.

 Overall, this is a thoughtful and promising study that is likely to make a meaningful contribution to both implementation science and mental health research. with modest refinements the protocol will provide a robust foundation for a high-quality realist review.

Is the study design appropriate for the research question?

Yes

Is the rationale for, and objectives of, the study clearly described?

Yes

Are sufficient details of the methods provided to allow replication by others?

Partly

Are the datasets clearly presented in a useable and accessible format?

Yes

Reviewer Expertise:

mental health research

I confirm that I have read this submission and believe that I have an appropriate level of expertise to confirm that it is of an acceptable scientific standard.

# Response

**Published:** 2026-03-16

**Authors:** Sean Paul Teeling

Dear Dr Desmet

 Sincere thanks for the constructive suggestions provided. These comments were extremely helpful in strengthening the clarity and methodological transparency of the study. In response, several revisions have been made throughout the manuscript.

 Additional detail has been added to clarify the composition and roles of both the Local Reference Group and the Expert Panel. These sections now outline the inclusion of MHFA programme participants, accredited MHFA instructors involved in programme delivery, and staff responsible for organisational wellbeing initiatives within hospital settings, alongside academic researchers with recognised expertise in realist methodology and organisational wellbeing (Lines 81–84; 93–98).

 The introduction has also been revised to provide a clearer definition of staff wellbeing within hospital contexts, specifying that it refers to psychological wellbeing and related workplace experiences such as burnout-related strain, psychological safety, mental distress and perceived organisational support. Workplace culture is clarified as a contextual influence that may shape how programme mechanisms operate, rather than as a standalone outcome (Lines 11–16).

 In response to your observation that outcomes were framed too narrowly in managerial or metric-driven terms, the outcomes section has been revised to distinguish between proximal, intermediate and distal outcomes. This layered structure supports realist programme theory development by recognising that interventions may produce different types of outcomes across stages of implementation and organisational influence (Lines 125; 127–132).

 Your observation regarding the rigidity of the original PICO structure was also particularly helpful. The manuscript now clarifies that PICO is used only to structure the initial search strategy and that the review will follow an iterative, theory-led searching process consistent with realist methodology, allowing further purposive searches to explore emerging contextual and mechanistic explanations as programme theories develop (Lines 133–138). In addition, the restriction to acute hospital settings has been relaxed to allow inclusion of studies from adjacent healthcare contexts where they provide transferable explanatory insight relevant to hospital staff wellbeing (Line 125).

 To further support methodological clarity, a brief illustrative Context–Mechanism–Outcome example has been added to the introduction to demonstrate how provisional programme theories may be articulated and refined during the review process (Lines 43–47).

 Finally, a reflexivity statement has been included in the methods section outlining the research team’s background in healthcare improvement and organisational wellbeing research and describing the reflexive processes that will be used to support interpretive rigour, including team discussion, expert panel adjudication and stakeholder engagement during programme theory refinement (Lines 203–214).

 All revisions have been highlighted in yellow in the revised manuscript and the relevant line numbers have been provided above to assist the reviewer in locating the changes. We are grateful for your constructive feedback, which has helped strengthen the explanatory focus and methodological coherence of the protocol and ensures that the review is positioned to generate programme theories that can inform how and under what conditions MHFA may contribute to staff wellbeing across healthcare settings.
